# Medical and Philosophical Intelligence

**Published:** 1817-01

**Authors:** 


### MEDICAL AND PHILOSOPHICAL INTELLIGENCE.

THE following paper being transmitted to us without a name is not to be considered as a document, but as the statement of a case, for which it is doubtful whether the Act has made sufficient provision.

*To the Master, Wardens, and Court of Assistants, of the Society of Apothecaries.*

GENTLEMEN,

HAVING been appointed by you, a member of the Court of Examiners of the Society of Apothecaries, and a circumstance having occurred, in the execution of that duty, which renders an appeal to the Court of Assistants imperative upon me, I beg leave to state the case for your consideration and decision.

A candidate of the name of A. B. applied, on the 12th ultimo, to the Court of Examiners to be examined for a certificate to practise as an apothecary. He stated that he had been bound apprentice, as a citizen and ironmonger, in the year 1807, for seven years, to a Mr. C. D., chemist and druggist, of ...... street, London; that his indenture was in the Chamberlain's Office; but that his master had given him the following certificate, which he produced:

"I hereby notify to whom it may concern, that A. B. was bound apprentice to me on the 3d day of November, 1807, for the term term of seven years; that, during the said period, he conducted himself to my satisfaction; that he was in the habit of dispensing medicines, and making preparations in pharmacy, under my directions; and that he is of good moral conduct.

"*London; June* 18, 1816. (Signed) C. D."

Half the members of the court (including myself) objected to Mr. A. B. being admitted to examination, upon the ground that he had not served an apprenticeship to an apothecary as the Act directs; and, therefore, could not claim under the Act to be examined. The casting vote decided that he should be admitted to examination. I then offered a formal and written dissent, founding my objection on its being an illegal proceeding. A proposal to postpone deciding the question of examination for a week, was also rejected. As I considered the proceeding as contrary to law as it was to policy, I refused putting my signature to a certificate granted under such circumstances; and the reasons that influenced my determination, are:—1st, That the candidate had served his apprenticeship to a chemist and druggist, and not to an apothecary. 2dly, That the master's certificate did not set forth, or assume, that he was an apothecary, but a mere dispenser of medicines. 3dly, That a dispensing chemist and druggist, who neither before nor since the passing of the Act ever called himself, or wrote upon his house, *apothecary*, is not an apothecary, nor can his apprentice claim as such. 4thly, Because, whatever doubts might have prevailed formerly, as to who were, or who were not, apothecaries, none can exist now; since the committee of the House of Commons would not grant the prayer of the petition for the late Act, until evidence had been examined, that apothecaries had, from time immemorial, visited and prescribed for the sick; and, unless satisfactory proofs of that fact had been adduced before them, the Act which expressly enjoins the Court of Examiners to examine all apothecaries touching "their skill and abilities in the science and practice of medicine," (see section 14,) would never have passed. 5thly, That the said master is not an apothecary is also clear, according to the usage of the society; for his shop is exempt, by former custom, as well as by the Act itself, from the inspection of the society's examiners; nor has it ever been examined by the College of Physicians, although it has remained in its present state for thirty years past. 6thly, That no apprentice who had served this master, or any other chemist and druggist, would be admitted to purchase his freedom of the society; which, if I am rightly informed, always require indentures of apprenticeship to a member of the society, or to a regular apothecary. 7thly, The chemists and druggists opposed the Bill, and procured the introduction of a special clause, (see Section 28,) to free them from being considered apothecaries, and from being subjected to the provisions of the Act. 8thly, That the Court of Assistants have themselves sanctioned the opinion, that dispensing druggists and chemists were not apothecaries. 9thly, That the Court of Examiners have themselves also decided, that persons serving an apprenticeship to chemists and druggists druggists solely, have no right to be examined, or to practise as apothecaries. Lastly, If it be established, that dispensing chemists and druggists are legal apothecaries, the Court of Assistants cannot, by virtue of any bye-law, refuse any chemist or druggist admission into the society, after having received a certificate as a qualified apothecary. Thus a precedent might be introduced, that may prove subversive of the society itself as a body of apothecaries.

These, gentlemen, are not merely my arguments, but they embrace the opinions of the best legal authority I have been able to consult. But, as the legal opinions to which I allude could not be received, as they did not come through the official and regular channel, I submitted a motion at the last meeting of the Court of Examiners, that the Court of Assistants should be requested to take counsel's opinion on a subject so delicate and important, in order that the minds of all the examiners might be correctly informed, and the consciences of the majority of them be satisfied, whether they are acting right or wrong; but this question was also negatived, by a casting vote only. Failing, therefore, in my attempt to lay this case before your court in the more consistent way, my only resource is, to make a personal appeal for advice to guide my future conduct on any similar occasion. If the law be otherwise than I have been taught, and interpret it, however much I may lament the defect in the Act, it is my duty to submit, and conform to its enactments; but, having at present a quite opposite impression, I feel it equally my duty, as a member of the Court of Examiners, until instructed by legal authority to the contrary, to firmly resist, by every fair means, all attempts to admit candidates, having served dispensing chemists and druggists only, to an examination; neither will I sign the certificate of any such candidate. I, therefore, most respectfully solicit from the Court of Assistants an early intimation of their pleasure on the subject now proposed to them.

I have the honor to be, gentlemen,

Your obedient humble servant, &c.

*October* 2, 1816.

---

*Extract of a private Letter from Masham,* near *Bedale.*—A very eminent medical practitioner in this place, and 20 miles round about it, has recommended, with universal success, the following method of rendering *very* unsound corn capable of being made into perfectly good bread.—Add to eighteen pounds of unsound flour, or meal, two ounces of salt of tartar, dissolved in warm milk and water, or only warm water, and let the whole be kneaded very stiff. If yeast should be used, after the said liquid, and the meal has been well mixed, the dough should be set before a fire for an hour, in the same manner as common bread. As there are degrees of unsoundness in corn, Mr. Baines suggests, that the said quantity of salt of tartar might be used with sufficient effect with twenty-eight pounds of flour, or meal, where the unsoundness is not considerable.

I have troubled you with the above communication, as I am fully fully convinced of its importance in alleviating the unfortunate scarcity which at present threatens to afflict the country; and from being persuaded that you may contribute very materially to recommend so useful a receipt to general use where wanted.

---

The following is the postscript of a letter much too long for insertion:—" P. S. Since I commenced the above letter, I have been gratified by the perusal of an article in the Quarterly Review for November 1816, (to which I beg to refer your readers, as containing many judicious and valuable remarks on the subject of insanity and madhouses,) in which the writer, although far from entirely concurring in sentiment with Mr. HILL, expresses a very different opinion, in reference to the Treatises extant upon Insanity, from that which you have thought proper to express in your Journal.—You say, as already quoted, " Mr. HASLAM's performance is the only one which offers any insight into the mode of treatment."—The writer of the article referred to, on the contrary, considers Mr. HILL's as the best systematic work on insanity which has yet been presented to the public. (Vide Quarterly Review for 1816, page 401.)"

We cannot help being pleased with H.'s zeal in behalf of his friend, but he must be aware that such long communications would occupy more space than our readers expect by way of explanation, and from anonymous writers. It is probable that the remark concerning Mr. HASLAM's book was written by a fellow-labourer who had not seen Mr. HILL's. In our review of Mr. HILL, it is clearly shown that he enters on the TREATMENT of Insanity in all its forms.

---

Mr. BRAND is appointed Secretary to the Royal Society, in the room of Dr. Woolaston, who has resigned.

---

At the meeting of the London Medical Society, on the 9th ult. a letter from Dr. MEASE, of Philadelphia, was read, containing an account of the death of the late Dr. Rush. By this it appears, that the doctor was right in his opinion of his own disease (pleurisy), and probably fell a sacrifice to his ill-timed modesty.—On the same evening, Mr. STEVENSON exhibited and explained to the members present the mechanism and mode of using several instruments which he has invented, and finds very convenient for performing different operations upon the eye and ear. The attention of the members was particularly attracted by the simplicity of an admirably-contrived pair of ear-forceps, serving equally for the extraction of polypi or any extraneous substances from the meatus auditorius externus, and as a speculum auris. A scarificator, which is alike applicable for drawing away blood from the internal membrane of the eye-lids, and membranous lining of the auditory passage, when labouring under high inflammatory action, surpasses any apparatus of the kind in common use, by its elegance and neatness, but most of all its apparent adaptation to the purposes for which it is designed. The superior advantages of a knife, contrived for making with greater safety and effect the section through the cornea, preparatory to the removal of the opake lens, was described in a very satisfactory manner. Lastly, a small spear-shaped lancet, fixed in an hexagonal handle, was represented, as not only well suited for making a puncture into the cornea, in order to evacuate the contents of the aqueous humour in certain diseased states of the organ of vision, but likewise with the aid of a grooved conductor, made to correspond with the size of the instrument, for the removal of grumous blood, pus, or the broken fragments of cataracts from the anterior chamber of the eye. These, which, as far as we recollect, constitute the whole of the instruments shown by Mr. Stevenson, do great credit to the inventor, and promise to facilitate operations on that delicate organ.

---

It is with real concern we feel ourselves obliged to advert to some events in the London Hospital, the full history of which we shall not pretend to develope. On the resignation of Mr. Thomas Blizard, three candidates presented their pretensions to the governors: Mr. Armiger, Mr. Frampton, and Mr. Andrews. All had the customary claims of apprenticeship to the hospital; and the priority of this claim stands, we believe, in the order in which we have placed them; but the votes of the governors have been in the inverse ratio of that order. The influence of the Blizards has been often complained of in that house; yet the very large majority by which Mr. Andrews was chosen, is more than we can entirely place to the account of that influence. Mr. Frampton complains that he has paid 900l. to the hospital; but, surely, he would not wish to claim the surgeoncy as a purchase. As to the apprentice-fee, we can only regret that the friends of so many young men are induced to give these large sums on such uncertain prospects; nor can we help expressing our surprise, that hospital-surgeons should receive so many apprentices, when so few can succeed them.

Another expense of which Mr. Frampton complains, is the purchase of the anatomical school. That this school was raised at some expense by Sir William Blizard, cannot be questioned; and, though it does not pay the teacher like other establishments in better situated hospitals, yet it must always prove an advantage to every young surgeon, and surely much more than return interest and principal for any charge which may attend the first introduction of a teacher.

Having said thus much, we wish not to be supposed competent to offer any opinion on the claims of the different candidates; but, as the public at large must be still worse judges, we always regret every appeal to them. If we may be allowed an opinion, we suspect it is this very uncertain judgment (for in medicine more than in any other science we may say *judicium vulgi fallax*) of the public, that has been the source of Mr. Frampton's failure. Probably, whilst he has been passing his time in the dissecting-room, and trusting to a supposed compact, which cannot be proved, Mr.

Mr. Andrews and his friends have been strengthening their connection with the governors. We mention this, not to encourage that kind of tortuous introduction to such honours, but to remind young men that, however great their merits may be, the public must be attended to; that it is not enough that promises are presumed; there must be a perfect understanding; and even that should not depend entirely on the parties themselves.

---

*Prize Memoir on Hydrophobia.*—The union of physicians at Paris, under the title of The Medical Circle, wishing to acquire new light on the nature of rabies, or canine madness, (*la rage*,) offers a gold medal of the value of 300 francs, for the best Essay on the Question, including the following points:

1. In what consists the disease known under the name of Hydrophobia, (*la rage*)?
2. What are the characteristic symptoms of it in men and animals?
3. Whether there are examples of its developing itself spontaneously in men?
4. Whether there exist several species, and what are those species?
5. Whether they are contagious for man, and in what manner the contagion is communicated?
6. Whether we ought to attribute the accidents which follow the bite of enraged animals to a particular virus, to the nature of the bite, or to the physical lesion of the bitten parts, or to terror?
7. Whether the fluids or solids present any alteration peculiar to this disease, during life or after death?
8. What is the best mode of treatment, either as prevention or cure?

The memoirs must be written in French or Latin, and bear, as usual, a motto, to be repeated on a sealed letter, containing the author's name, and addressed (post paid), before the 1st of January, 1817, to Dr. Chardele, General Secretary of the Medical Circle, No. 23, Rue Cassette, Paris. The Prize will be awarded in a public extraordinary sitting, to be held in the month of March, 1817.

---

### *Pharmaceutic Improvements.*

*On New Preparations of Ipecacuanha, Peruvian Bark, and Rhubarb;* by M. Coldefy, Apothecary at Crepy.—To render the administration of these remedies the more agreeable to the most delicate children, M. Coldefy has proposed a mode of dissolving the active principles in alcohol of 20 degrees, and then combining this alcohol with sugar.

*Tincture of Ipecacuanha.*—Ipecacuanha in coarse powder, ℥v. Alcohol of 20 degrees, ℥xii. Infuse in a moderate heat six days, decant, and pour into it alcohol of 20 degrees, ℥vi. Leave it to infuse eight days, decant, and add alcohol of 20 degrees, ℥vi. Infuse for eight days more, filter, and mix the three liquors, which will be about twenty-three ounces; pour in the residue placed in the filter what is wanting of alcohol of 20 degrees, to make up twenty-five ounces of tincture.

*Sugar of Ipecacuanha.* — ℞. Of the above tincture, ℥ii½. White dry sugar in powder, ℥ii. Mix them carefully in a wide earthen pan, evaporate to dryness, taking care to stir the mixture twice or thrice a day with an ivory spatula. The operation will be completed the third day; triturate the product gently in a marble mortar, and, as it granulates, pass it through a coarse hair sieve, and preserve it in a phial well corked. The granulated form appears to me the most eligible.

It will be apparent from this formula, that four ounces of sugar are used to one ounce of ipecacuanha; the quantity of sugar may appear too great, but it has been preferred to a less proportion, because various experiments have convinced us, that, when the sugar is in too small a quantity, the medicament is not so perfect when dissolved in water; besides, it possesses a more agreeable flavour, and produces precisely the same effect.

*Tincture of Peruvian Bark.*—℞. Selected bark in coarse powder, ℥viii. Alcohol of 20 degrees, ℥xx. Let it infuse in a gentle heat, decant carefully, and add of alcohol, of 20 degrees, sixteen ounces. Infuse in the same temperature eight days more, decant carefully, and pour on the residue alcohol, of 20 degrees, twelve ounces. Infuse eight days, decant, and add of alcohol 12 degrees; let it infuse six days, filter, and pour on the residue to make up fifty-six ounces of tincture. After having thus obtained a strong tincture, pour it into a small sand-bath, with sixteen ounces of water, at the temperature of 60 degrees Reaumur; place the sand-bath in its cucurbit, and keep up the same heat for six hours, taking care to cover it closely, decant the liquor, and pour on the residne 11 oz. of water at 60 degrees; proceed as above, express the whole of the fluid, and mix the two infusions, which ought to weigh eighty-four ounces.

I have subjected the bark to the latter observation, in order to separate the kinate of lime, which is insoluble in alchohol, and the little extractive matter which might have resisted that agent, which is thus perfectly obtained.

*Sugar of Peruvian Bark.*—℞. Of the above tincture, ℥vii. White dry sugar in powder, ℥iii. Mix in an earthen vessel, and place on a stove. Take aqueous infusion of bark, ℥xii. Sugar, ℥iv. Mix in an earthern vessel, and treat it like the ipecacuanha; unite the products, triturate and granulate them as above. The bark thus treated yields one ounce, two drachms, and a half, of a gum resinous extract, or nearly one-third of its weight.

I have treated Moscow rhubarb with alcohol of 20 degrees; four infusions extracted all the virtue, it lost nearly one-half. I have obtained by the same method, a sugar possessing all the virtues of the root. The liquid must, on each occasion, be expressed from the root.

On

*On the Honey of Mount Hymettus, by* M. Cadet.—We only know the honey of Athens, or Mount Hymettus, by the praises which the Greeks and Romans bestowed upon it. The ancients, being ignorant of crystallized sugar, paid their attention entirely to honey, which was estimated according to its mildness. The Latin authors boast of the honey of Mount Hybla, in Sicily, that of Carina, &c. &c. in particular, that of Mount Hymettus, near Athens, is that mentioned by Martial, Lib. vii. Epig. 87.

*Pascat et Hybla meas, pascat Hymettas apes.*

Horace sung it also in ridiculing a voluptuary who refused to drink the Falernian wine unless softened by the honey of Hymettus;

*Nisi Hymettia mella Falerno*
*Ne biberis diluta.*—Lib. ii. Sat. 5. v. 15.

Silius Italicus, l. ii. v. 218, also cites it:

*Sparsa super flores examina tollit Hymettos.*

This honey has lost nothing of its reputation: the modern Greeks send it to Constantinople, where it is highly esteemed, and where it is called *Cosbachi.* They have observed that it bears more water when used for sherbet or hydromel. The *Calayers* are astonished that Narbonne honey is esteemed on account of its whiteness, that being with them a proof that the honey was not perfectly made by the bees. Strabo says, the best honey is made without vapour, and therefore the Greeks do not destroy the bees, however old they are, as is the practice in some countries. The honey of Mount Hymettus is generally supposed to be indebted for its excellence to the odoriferous flowers which grow on Mount Hymettus. Pliny ascribes it to the thyme that grows there, which he tells us was transported to Italy in the hope of obtaining similar honey, but the experiment did not succeed.

This opinion of the Roman naturalist is very singular, as the honey of Athens is not odoriferous, is a beautiful yellow colour, mixed with a little wax: but, since the refinement of sugar of the cane and beet-root, the natural product celebrated by Homer, and prized so highly by the masters of the world, has lost its grand interest as an article of luxury.

---

*Sulphurous Fumigations.*—The success of sulphurous fumigations in chronic disorders, the itch, &c. has induced the French government to order to be printed at the Royal Printing-office, a Memoir of Dr. Gales on the subject, detailing the practice as established by him at his own house at Paris.

The ready, easy, and economical cure of cutaneous and other disorders by the application of this method is detailed in three official reports commanded by the Minister of the Interior. The first, of the medical jury of the civil hospitals; the second, a committee of the faculty of medicine; and the third, of the principal physicians of the capital: by which it is proved, that it is a certain and speedy cure for the itch, and generally in all chronic affections of the skin, and is a powerful auxiliary in gout and and rheumatism. It has also effected astonishing cures in paralysis, though its effect is not always certain in this disorder. The Duke de Richelieu and Count de Vaublain, ministers for foreign affairs and the interior, have attended the curative process; and the minister of the interior has written to all the prefects, or governors of provinces, desiring them to adopt the method of Dr. GALES in all hospitals, workhouses, houses of correction, &c. and that Dr. GALES, though as the inventor of the process, and consequently has the exclusive right of practising, yet, on the demand of the prefects, he will supply for these benevolent and public purposes the necessary apparatus for 20l.

The Memoir, and the three Reports, are of great importance: having only just appeared, we cannot notice them properly this month, but shall give an article on the subject in a future Number.

---

*Physicians condemned to Celibacy.*—Formerly in France, the "Professors of the Faculty of Medicine" were *clerks*, and condemned to celibacy; but they made such pressing solicitations to Cardinal D'Estouteville, in 1452, and represented to him in such strong terms the temptations to which they were unceasingly exposed, that they at length obtained permission to marry.

---

*Middlesex Hospital.*—Dr. P. M. LATHAM and Dr. SOUTHEY will commence a Second Course of Lectures on the Practice of Physic, at this hospital, on Monday the 3d of February next.

Dr. ADAMS will begin his Spring Course of Lectures on the first Tuesday in February, at ten o'clock, at his house in Hatton Garden.

*Theatre of Anatomy.*—Mr. TAUNTON's Winter Course of Lectures on Anatomy, Physiology, Pathology, and Surgery, will commence on Saturday, January 18th, 1817, at eight o'clock in the evening precisely, and be continued every Tuesday, Thursday, and Saturday, at the same hour.

Mr. CLARKE will commence his next Course of Lectures on Midwifery and the Diseases of Women and Children, on Monday, January 27th. The lectures are read every morning, from a quarter-past ten to a quarter-past eleven, for the convenience of students attending the hospitals.

*Theatre of Midwifery*, 68, *Berners-street.*—Dr. CLOUGH, Physician-Accoucheur to the Endeavour Society, to the St. Mary-la-bonne General Dispensary, and to the Benevolent Institution for the Delivery of Poor Women at their own Habitations, &c. &c. commences his Winter Course of Lectures on the above Science, &c. on Monday morning, the 6th of January, at half-past ten.

---

*Our* REPORT ON DISEASES *is deferred till the next Number, when we shall offer our Remarks on the Yearly Bill of Mortality.*

METEOROLOGICAL

### METEOROLOGICAL REGISTER.

### *From November the 25th, to December the 25th, 1816.*

**Kept by C. BLUNT, Philosophical Instrument Maker, No. 38, Tavistock-Street, Covent-Garden.**

| Moon. | Day. | Wind. | Barometrical Pressure. Max. | Min. | Mean. | Temperature. Max. | Min. | Mean. | |
|---|---|---|---|---|---|---|---|---|---|
| ◐ | 26 | SE | 30·02 | 30·03 | 30·25 | 49 | 42 | 45·05 | Fair |
| | 27 | E | 30·04 | 30 04 | 30·04 | 48 | 43 | 45·05 | Fair |
| | 28 | E | 30·48 | 30·48 | 30·48 | 46 | 44 | 45· | Fair |
| | 29 | NE | 30·48 | 30·04 | 30 44 | 43 | 37 | 40· | Fair |
| | 30 | NE | 30 75 | 30·75 | 30·75 | 40 | 31 | 35·05 | Fair |
| | 1 | NW | 30·78 | 30 07 | 30 74 | 37 | 32 | 34 05 | Frost |
| | 2 | NW | 30 64 | 30·06 | 30·62 | 40 | 35 | 37·05 | Fair |
| | 3 | W | 30·55 | 30 55 | 30·55 | 40 | 33 | 36·05 | Fair |
| ○ | 4 | W | 30·57 | 30· | 30·28 | 45 | 37 | 36· | Fair |
| | 5 | W | 30· | 30· | 30· | 39 | 35 | 37 05 | Fair |
| | 6 | NW | 29 06 | 29 06 | 29·06 | 40 | 36 | 38· | Rain |
| | 7 | W | 29·55 | 29 57 | 29·56 | 42 | 37 | 39 05 | Fair |
| | 8 | W | 29 58 | 29·54 | 29 56 | 41 | 38 | 39· | Rain |
| | 9 | NW | 29·58 | 29 54 | 29 56 | 47 | 39 | 43· | Rain |
| | 10 | W | 29·05 | 29 05 | 29·05 | 42 | 38 | 40· | Fair |
| | 11 | SW | 29·48 | 29·46 | 29·47 | 42 | 38 | 40· | Fair |
| ◑ | 12 | SW | 29·45 | 29·45 | 29 45 | 50 | 44 | 47· | Rain |
| | 13 | NW | 28·81 | 28·81 | 28·81 | 51 | 46 | 48· | Rain |
| | 14 | NW | 29 15 | 29·10 | 29·12 | 50 | 45 | 47·05 | Rain |
| | 15 | NE | 29· | 29 | 29· | 41 | 34 | 37·05 | Fair |
| | 16 | NE | 29·20 | 29·10 | 29·15 | 43 | 33 | 38· | Fair |
| | 17 | NE | 29 05 | 29· | 29.25 | 41 | 30 | 35·05 | Rain |
| ● | 18 | NE | 29·26 | 29·26 | 29·26 | 37 | 30 | 38·05 | Fair |
| | 19 | NE | 29·68 | 29 60 | 29·34 | 38 | 30 | 34· | Snow |
| | 20 | E | 30·44 | 30·44 | 30·44 | 35 | 26 | 30·01 | Frost |
| | 21 | E | 30·61 | 30 31 | 30·46 | 31 | 24 | 27· | Frost |
| | 22 | SE | 30 64 | 30 64 | 30·64 | 30 | 21 | 25·05 | Frost |
| | 23 | S | 30·05 | 30·05 | 30·05 | 46 | 39 | 42·05 | Rain |
| | 24 | SE | 30·01 | 30· | 30·05 | 48 | 40 | 44· | Fair |
| | 25 | SE | 29·71 | 29· | 29·36 | 46 | 40 | 43· | Fair |

### RESULTS.

| | | | | | |
|---|---|---|---|---|---|
| Mean barometrical pressure of the month | | 29 80 | Mean temperature of the month | | 39 deg. |
| Maximum 30 78, | wind at | NW | Maximum 51, | wind at | NW |
| Minimum 28 81, | —— | SW | Minimum 21, | —— | SE |

*Scale exhibiting the prevailing Winds during the Month.*

| N | NE | E | SE | S | SW | W | NW |
|---|---|---|---|---|---|---|---|
| 0 | 7 | 4 | 4 | 1 | 2 | 6 | 6 |

| | Mean barometrical pressure. | Mean temperature. |
|---|---|---|
| From the first quarter on the 26th Nov. to the full moon on the 4th Dec. | 30 50 | 39 55 |
| —— the full moon on the 4th, to the last quarter on the 12th | 29·33 | 43 3 |
| —— the last quarter on the 12th, to the new moon on the 18th | 29 30 | 41 7 |
| —— the new moon on the 18th, to the first quarter on the 26th | 30· | 35·57 |

BOOKS

